# Long-Lasting Cranial Nerve III Palsy as a Presenting Feature of Chronic Inflammatory Demyelinating Polyneuropathy

**DOI:** 10.1155/2015/769429

**Published:** 2015-04-16

**Authors:** Rossella Spataro, Vincenzo La Bella

**Affiliations:** ALS Clinical Research Center, Bio.Ne.C, University of Palermo, 90129 Palermo, Italy

## Abstract

We describe a patient with chronic inflammatory demyelinating polyneuropathy (CIDP) in which an adduction deficit and ptosis in the left eye presented several years before the polyneuropathy. A 52-year-old man presented with a 14-year history of unremitting diplopia, adduction deficit, and ptosis in the left eye. At the age of 45 a mild bilateral foot drop and impaired sensation in the four limbs appeared, with these symptoms showing a progressive course. The diagnostic workup included EMG/ENG which demonstrated reduced conduction velocity with bilateral and symmetrical sensory and motor involvement. Cerebrospinal fluid studies revealed a cytoalbuminologic dissociation. A prolonged treatment with corticosteroids allowed a significant improvement of the limb weakness. Diplopia and ptosis remained unchanged. This unusual form of CIDP presented as a long-lasting isolated cranial nerve palsy. A diagnostic workup for CIDP should therefore be performed in those patients in which an isolated and unremitting cranial nerve palsy cannot be explained by common causes.

## 1. Introduction

Chronic inflammatory demyelinating polyneuropathy (CIDP) is an autoimmune disease that targets the myelin sheath of peripheral nerves. The clinical picture is heterogeneous and is often characterized by a progressive or relapsing motor and/or sensory dysfunction in more than one limb of peripheral nerve nature [1, 2].

CIDP develops over at least two months, and its diagnosis is mainly based on physiologic and cerebrospinal fluid (CSF) studies [1]. The response to intravenous immunoglobulins, corticosteroids, and other immunosuppressants is also a key feature of CIDP.

The recent EFNS/PNS diagnostic criteria for CIDP have been validated, showing 81% sensitivity and 96% specificity [1].

Predominant cranial nerve (CN) involvement is a relatively unusual feature of CIDP [3–5], being described in only 5% of patients in a case series [3]. Oculomotor nerves (III, IV, and VI) are most often affected, followed by the CN VII and, more rarely, CN IX, X, and XI. A report showed that an isolated CN III deficit was a presenting feature of CIDP, two years before the onset of the symmetric polyneuropathy [6].

Here, we describe a case with a similar presentation, in which a nonreversible adduction deficit and ptosis in the left eye preceded by several years the onset of the polyneuropathy.

## 2. Case Report

A 52-year-old unmarried man referred to our Neurology Ward with years-long history of ptosis, adduction deficit in the left eye, and mild diplopia followed by slowly progressive sensory deficits, fatigue, and weakness in the lower limbs. More recently a bilateral foot drop appeared (more pronounced in the right foot) making the walking very difficult.

The onset of ptosis and diplopia was dated back to 14 years whereas the sensory symptoms and weakness appeared some seven years earlier. For several years the patient did not seek medical advice. In the past two years he underwent a brain and spine MRI (both negative) and electromyography/nerve conduction studies which showed reduced conduction velocity and bilateral and symmetrical sensory and motor involvement in the four limbs. A diagnosis of motor-sensory polyneuropathy of unknown cause was made.

The patient is an administrative officer and had never been exposed to chemicals, pesticides, neurotoxicants, and heavy metals. He is neither diabetic nor hypertensive. The family history is negative for hereditary motor-sensory polyneuropathies (HSMN). The neurological examination showed moderate hypotrophy in the four limbs distally, more prominent in the lower limbs. Walking was difficult because of a bilateral foot drop. Muscle tone was normal, and tendon reflexes were diminished in the upper limbs and absent in the lower limbs. Vibratory sensation was impaired in the lower limbs.

**Figure 1:**
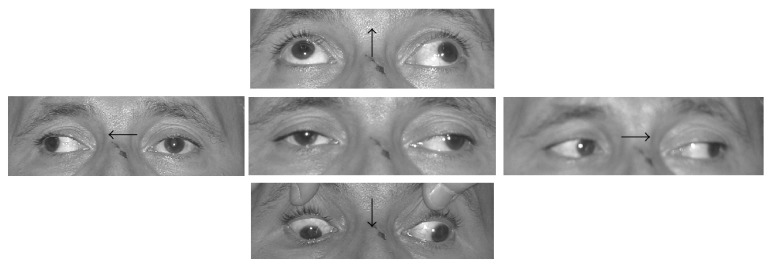
The left eye adduction deficit and ptosis in the patient. Note the slight ptosis also in the right eye. The left CN III deficit persisted unmodified after treatment with IVIg and methylprednisolone.

Visual acuity was 20/20 in both eyes. He had ptosis in the Oculus Sinister (OS) with a nearly complete adduction deficit. A slight ptosis without other abnormalities was also evident in the Oculus Dexter (OD) (Figure 1). Pupils had equal size in dim illumination and symmetric light reaction.

An extensive biochemical and immunological workup was performed that did not disclose abnormalities. In particular, anti-ganglioside antibodies (GM1, GM1b, GQ1b, GD1a, GD1b, and GT1b) and antimyelin-associated glycoprotein were negative. CSF analysis showed a cytoalbuminologic dissociation with one white cell per mm^3^ and a protein of 82 mg/dL. No oligoclonal bands were detected. Electroneuromyography demonstrated reduced conduction velocity with bilateral sensory and motor involvement (Table 1). Brain MRI and MR angiography were also performed but did not show abnormalities. Extensive genetic testing for HSMN I, including PMP22 duplication and connexin 32 mutations, gave negative results.

According to the European Federation of Neurological Societies/Peripheral Nerve Society Guideline on management of chronic inflammatory demyelinating polyradiculoneuropathy [1], a clinical diagnosis of clinically probable CIDP was made.

The patient was treated with IVIG (0.5 g/Kg/day for five days) followed by oral methylprednisolone at a dose of 8 mg/day for several weeks [8]. Muscle strength slightly improved, but ptosis and the adduction deficit were left unchanged.

## 3. Discussion

Cranial nerve involvement is relatively uncommon in CIDP [3, 5, 9]. Ocular motor involvement is characterized in most cases by an unilateral and not reversible deficit, often preceding the limb signs and symptoms by a short interval [3, 4, 6, 9].

In a case described, a CN III paresis occurred two years before the onset of the limb weakness and fatigue [6]. Furthermore, an MRI study demonstrated a bilateral enhancement of CN V and III, suggesting a more widespread cranial nerve damage [6].

Our case adds to this very limited literature, as it shows a very long interval (i.e., at least seven years) between the onset of the adduction deficit and ptosis of the OS and the appearance of the first symptoms of the motor-sensory polyneuropathy. Interestingly, the clinical inspection revealed a slight and isolated ptosis also in the right eye, suggesting a bilateral involvement of CN III. Subclinical or mild cranial nerve deficits are in fact relatively common in CIDP [1, 3, 4, 6].

Our patient for several years did not pay attention to his diplopia and left ptosis and sought for a doctor only after the appearance of the limbs weakness. He did not even notice the mild ptosis in the OD. Over such a lengthy period of time (i.e., fourteen years), the left ophthalmoparesis never showed an appreciable remission, and our neurophysiological, biochemical, immunological, and imaging studies excluded common causes of CN III palsies (e.g., diabetes, hypertension, viral infections, and intracranial aneurysm). Thus, the diagnosis of CIDP appeared the most plausible, linking the cranial nerves III deficits and the chronic polyneuropathy.

Anti-ganglioside antibodies, including the IgG anti-GQ1b gangliosides, which have been found to be associated with the acute ophthalmoplegia in the Miller-Fischer syndrome and in Guillain-Barré syndrome [10], were absent in our case. Lack of anti-ganglioside antibodies has been already described in patients with CIDP and ophthalmoplegia [4, 6]. Thus, our results further support the hypothesis that other antibodies might be involved in CIDP with ocular motor palsies [6].

In conclusion, we have described a variant of CIDP where a persistent ophthalmoparesis was preceding by years the appearance of the motor-sensory polyneuropathy. CN III seems in fact to be particularly susceptible to a permanent damage due to an autoimmune aggression [4, 6].

Neurophysiological and CSF studies should always be performed in patients with unremitting ocular motor nerve palsy not explained by common causes.

## Figures and Tables

**Table 1:** Nerve conduction study.

Nerve	*A*	Decrease (%)	CV	Decrease (%)	DL	Increase (%)
R-median (M)	6.0 mV	14	40.1 m/s	24.1	3.5 ms	0.28
R-median (S)	16.0 *μ*V	58	43.3 m/s	22.9	3.2 ms	12
L-ulnar (M)	5.4 mV	5	36.0 m/s	32.8	3.9 ms	30.1
R-peroneal (M)	2.6 mV	55	27.5 m/s	38.1	6.1 ms	31
L-peroneal (M)	3.0 mV	50	38.8 m/s	12.6	5.1 ms	10.1
R-sural	3.3 *μ*V	80	32.4 m/s	28.3	4.3 ms	53

*A* = amplitude (CMAP or SAP); CV = conduction velocity; DL = distal latency. Data are also expressed as percent decrease or increase with respect to the normal values reported in [7].
